# Use of Gum Cordia (*Cordia myxa*) as a Natural Starch Modifier; Effect on Pasting, Thermal, Textural, and Rheological Properties of Corn Starch

**DOI:** 10.3390/foods9070909

**Published:** 2020-07-10

**Authors:** Shahzad Hussain, Abdellatif A. Mohamed, Mohamed Saleh Alamri, Mohamed A. Ibraheem, Akram A. Abdo Qasem, Syed Ali Shahzad, Ibrahim A. Ababtain

**Affiliations:** Department of Food Science & Nutrition, King Saud University, P.O. Box 2460, Riyadh 11451, Saudi Arabia; abdmohamed@ksu.edu.sa (A.A.M.); msalamri@ksu.edu.sa (M.S.A.); mfadol@ksu.edu.sa (M.A.I.); aqasem@ksu.edu.sa (A.A.A.Q.); syedalishahzad@gmail.com (S.A.S.); ababtain.ibr@gmail.com (I.A.A.)

**Keywords:** corn starch, gum *Cordia*, pasting, rheology, texture

## Abstract

Incorporation of hydrocolloid gums in native starches help to improve their pasting, thermal, rheological and textural properties along with improvement in the stability of starch gels. The use of *Cordia* gum is not widely studied as a starch modifier and this fact could make this study more interesting and unique. This study investigated the effects of the non-conventional hydrocolloid gum (*Cordia* gum) on corn starch properties. Corn starch and gum *Cordia* (GC) blends were prepared at different replacement levels (0%, 3%, 6%, 9%, and 12%). The effect of GC levels on pasting, thermal, rheological, and textural properties were evaluated using rapid viscoanalyzer, differential scanning colorimeter, rheometer, and texture analyzer. The presence of GC significantly increased starch gelatinization temperatures, enthalpies, peak viscosities, final viscosities, and setback viscosities. GC improved freeze thaw stability in starch. The shear rate (1/s) versus shear stress (σ) data of all samples fitted well to the simple power law model (R^2^ = 0.97–0.99). The control had the lowest flow behavior index (*n;* 0.17), which increased to (0.36–0.56) with increasing GC levels. The consistency index (*K*) of the starch-gum blends increased with increasing GC levels. The dominance of elastic properties over viscous properties was demonstrated by G′ > G″. The magnitudes of G′ and G″ increased with increasing GC concentration. The outcomes could help to use this modification method as an alternative to chemical and enzymatic modification with respect to cost, safety, less time consumption and less requirement of process modifications.

## 1. Introduction

Starch is abundantly stored as an energy reserve in different plant parts including tubers, roots, stems, and seeds. Starch is widely used in food industry as a gelling, stabilizing, and thickening agent depending on the specific properties [1,2]. The functional properties of starches may vary depending on their source, structure, grain size, grain shape, amylose content, chain length of amylopectin, and methods of extraction [3]. The native starch sometime is not suitable for food application because of its poor stability, lower viscosity, increased retrogradation, shear thinning, and syneresis during storage when compared to modified starches. Consequently, native starch is modified by physical, chemical, or enzymatic modifications. The modifications by these abovementioned methods sometimes cause safety issues, cost escalation, and longer modification time. Recently, the use of hydrocolloid gums as a starch modification method is getting attention from researchers because it is cheap, easy, safe, and natural compared to already in practice modification methods [4,5,6,7].

Hydrocolloid gums are hydrophilic molecules of high molecular weight and high viscosity when dissolved in water. The hydrocolloids are comprised of sugars like xylose, glucose, galactose, mannose, arabinose, and uronic acid. They are used in different food formulations as gelling, thickening, stabilizing, and foaming agents or to improve their water holding capacity, texture, palatability, and dietary fiber content [8,9]. They are used to modify the rheological and pasting properties of different starches at very low concentration. Several researchers have already studied the use of different hydrocolloids (guar gum, xanthan gum, linseed gum, okra gum, gum Arabic, and may others) in different starch (potato, sweet potato, rice, corn, wheat, sorghum, etc.) [10,11,12,13,14,15,16,17]. *Cordia myxa* L. (Assyrian plum or lasura), locally known as “Bumber” [18], can be a source of plant based gum sources. *Cordia* has high nutritive value, rich in antioxidants and flavonoids and is used as anti-inflammatory, antimalarial, cough suppressant, appetite suppressant and diuretic [19,20]. The *Cordia* gum is anionic polysaccharide having strong adhesive properties and is used as an emulsifier and tablet binder [21,22]. It is also used in bread as an anti-staling agent [23] and coating of pine nut fruits to preserve them from oxidation [24]. *Cordia* gum is reported to have 1.8 million Da molecular weight [25] and the main components of the polymer includes galactose (27%), rhamnose (21%), mannose (17%), xylose (11%), glucose (10%), arabinose (9.5%), and uronic acids (5%) [21]. The use of gums from *Cordia* has not been studied in detail for its starch modifying properties; therefore, the current study was carried out to evaluate the effect of gum *Cordia* on the pasting, thermal, and rheological properties of corn starch. The outcomes of this research provides insights on the use of this gum as an alternative to existing gum sources that are used in starch and baking industries.

## 2. Materials and Methods

### 2.1. Collection of Raw Material

The mature *C. myxa* fruits were picked from the local farm and stored at refrigeration temperature till further use. Corn starch (20.4% amylose content) was obtained from a local company.

### 2.2. Extraction of Cordia Gum

Collected fruits were thoroughly washed, steamed for 3 min to inhibit the enzymatic activity, and seed were removed to obtain the pulp. The pulp was blended with distilled water at 1:3 ratio for 1 min, filtered through muslin cloth and centrifuged at 10,000× *g* for 30 min. The pH of freshly extracted supernatant was recorded as 6.7. The collected supernatant was neutralized (using 0.1 N, NaOH), freeze dried, ground into fine powder (60 mesh), and stored in airtight jars in refrigeration till further use.

### 2.3. Preparation of Starch-Gum Blends

The gum *Cordia* (GC) freeze dried powder was blended (in triplicate, i.e., each blend was prepared 3 times) with corn starch at 3, 6, 9 and 12%. The blends were prepared on the dry weight bases by keeping in view the 9% initial moisture content of corn starch and 11.2% of gum *Cordia*. The wet blending method was used to achieve uniform distribution of gum in starch. The starch-gum blends were mixed with water (1:2), stirred well, and freeze dried. The freeze dried blends were milled to fine powder and stored in air tight jars till further use. The moisture content of the blends was recorded between 9–11% which was standardized in all experiments based on their respective procedure.

### 2.4. Thermal Properties of Starch-Gum Blends

DSC (Q 2000, TA instruments, USA) was used to study the thermal behaviors of starch-gum blends. Samples (6–8 mg) and 10 µL distilled water were added into aluminum pans. Pans were hermetically sealed and allowed to equilibrate at room temperature for 3 h. Empty pan was used as a reference cell. Thermal scanning of sealed samples was done from 30–120 °C @ 10 °C/min increment. Thermal transitions like enthalpy ΔH (J/g), onset temperature (OT), and peak temperature (PT) were determined using the Universal Analysis Software.

### 2.5. Pasting Behavior of Starch-Gum Blends

Starch-gum blends and native corn starch (3 g at 14% moisture basis) were transferred into aluminum canisters and distilled water was added to achieve a total weight of 28 g. The slurry was incubated at 50 °C for 30 s, heated to 95 °C in 4.40 min (at 10.23 °C/min), incubated at 95 °C for 4 min and finally cooled to 50 °C in 4.40 min (at 10.23 °C/min) and incubated at 50 °C for 2 min. The data for different parameters was processed using Thermocline window software provided with the instrument.

### 2.6. Freeze Thaw Stability of Gels

Starch gels were cooked (3 g starch, 14% moisture basis, dissolved in water to achieve total 28 g weight) using the rapid visco analyzer (RVA) program as mentioned in Section 2.5. Cooked starch gels were transferred into tubes and stored in the freezer at −20 °C. Frozen gels were taken out after 4 days and thawed in hot water bath at 50 °C for 30 min. Centrifugation was done at 3000× *g* for 15 min, and the water separated from the gels was measured. The gels were then stored in freezer for 4 days until another freeze thaw cycle. Separation of water from gels after 4 and 8 days storage was reported as % syneresis.

### 2.7. Textural Properties of Gels

Starch gels obtained from RVA experiment (3 g starch at 14% moisture) as described in Section 2.5 were kept in aluminum containers (35 mm height, 30 mm internal diameter) and stored at room temperature overnight. Brookfield CT3 Texture Analyzer (Brookfield Engineering Laboratories, Inc. Middleboro, MA, USA) fitted with 12.7 mm wide and 35 long cylindrical probe was used to compress the gels in two penetration cycles at a speed of 0.5 mm/s to a distance of 10 mm. Textural properties like gel hardness, springiness, cohesiveness, and adhesiveness were recorded directly while chewiness was calculated as a product of gumminess and springiness.

### 2.8. Flow Behavior of Starch Gels

The flow curves as a dependence of shear stress on shear rate were determined at 25 °C with shear rate of 0 to 300 (1/s) using TA Discovery Hybrid Rheometer (New Castle, PA, USA) equipped with cone-plate measuring system (2° angle, 40 mm in diameter). Starch/gum blends were cooked in RVA using same weight and experimental conditions as mentioned in Section 2.5. Cooked gels were transferred to plate and equilibrated at 25 °C for 1 min. Excess of the sample was trimmed off with spatula after adjusting the geometry gap to 100 µm. The experimental curves were described by power law model (Equation (1)).

(1) $$\tau =K\dot{\gamma^{n}}$$

where *τ* = shear stress (Pa), K = consistency coefficient (Pa s), *γ*˙ = shear rate (s^−1^), and n = flow behavior index (dimensionless).

### 2.9. Dynamic Shear Properties of Starch Gels

Dynamic rheology measurements were carried out on cooked starch samples obtained from RVA experiments (details provided in Section 2.5) using a DHR-Hybrid Rheometer. A similar geometry gap and cone plate system was used as mentioned in Section 2.8. Dynamic shear data was obtained at frequency sweeps ranging from 0.1–100 rad/s at 5% strain (within linear viscoelastic region). Experimental data was processed to calculate storage modulus (G′) and loss modulus (G″), and tan delta as a function of angular frequency.

### 2.10. Statistical Analysis

All data were collected in triplicates (for triplicated blends as mentioned in Section 2.3) and analyzed by analysis of variance (ANOVA). Duncan’s multiple range (DMR) at *p* ≤ 0.05 was used to compare means using SPSS (IBM Statistical Analysis Version 21).

## 3. Results and Discussion

### 3.1. Thermal Properties of Starch-Gum Blends

Thermal properties of starch blends determined using DSC is presented in Table 1. The onset temperature (OT), peak temperature (PT) and enthalpy of gelatinization (ΔH) of corn starch were significantly affected due to the presence of gum *Cordia* at various levels. The enthalpy of gelatinization (ΔH) gradually increased with gum presence up to 13.63 ± 0.10 J/g at 9% gum level, and reduced to 11.86 ± 0.06 J/g at 12% gum level. Concentration dependent response of gum was observed on the gelatinization enthalpy of starch. The ΔH of plain corn starch was 12.08 ± 0.19 J/g. The increase in ΔH due to the presence of gum could be attributed to hydrophilic nature of gums which binds the available water molecules and reduction in starch hydration, reduction in chain mobility and ultimately resulting in higher energy requirement for melting. Similar results were reported by Torres, et al. [26] where they observed increase in gelatinization enthalpy of chestnut starch when concentration of guar gum was increased. An increase in ΔH due to presence of xanthan gum in sweet potato starch, and *Mesona bloom* gum in rice starch [15,27] has similarly been recorded. The decrease in gelatinization enthalpy at 12% could be attributed to the fact that competition of water between gum and starch granules was much higher and availability of water in crystalline regions was reduced which ultimately resulted the partial gelatinization of these regions [14]. Another plausible reason could be starch dilution which resulted in reduction in amylose content in the blend in the presence of higher gum level because similar phenomenon was noticed as lower gel hardness and reduced flow index (*n*) of the power law at 12% gum concentration. The reduction in gelatinization enthalpies with higher levels of different hydrocolloids is well supported by the many other studies. The peak and onset temperatures of the gum blends were also significantly increased with the increase of gum *Cordia* percentage. The highest OT (71.34 ± 0.12 °C) and PT (77.66 ± 0.20 °C) were recorded in 12% blend while lowest OT (66.36 ± 0.15 °C) and PT (70.96 ± 0.52 °C) were noticed in plain corn starch (control). The presence of GC in the blends resulted in almost 7 °C increase in OT as compared to plain corn starch. The amount of energy required for reversible swelling o starch granules is called as onset temperature [14]. The increase in onset temperature could be attributed to different factors like lower ratio of free water to starch due to internment of water molecules by GC, reduction in heat transfer rate and mas transfer of water that resulted in difficulty of starch gelatinization in the presence of higher levels of gums [28,29]. Several researchers have reported similar increasing trend of PT when hydrocolloid gums were mixed with different starches [11,12,13,14,26]. The temperature recorded at the peak of gelatinization is called as peak temperature (PT) and it is called as moisture dependent property in starchy foods. The increase in peak temperature with higher levels of GC in the starch blends could be attributed to the gum ability to hinder water absorption by the starch granules and least water content resulted in higher peak temperature [10,30].

### 3.2. Pasting Behavior of Starch-Gum Blends

Pasting properties of starch-gum blends are presented in Table 2. Presence of gum in the blends had significantly (*p* ≤ 0.05) increased the peak (PV), final (FV), and setback (SB) viscosities while a slight decrease in peak time and peak temperature of blends was observed. The blend with 12% GC had the highest PV (3269 ± 33.23 cP), FV (3032 ± 15.56 cP), and SB (1695 ± 7.78 cP), while the lowest PV (2002 ± 24.04 cP), FV (1915 ± 39.60 cP), and SB (994 ± 2.83 cP) were recorded in control (plain starch). Peak viscosity is considered to be the point where maximum swelling of starch takes place. The increase in peak viscosity due to higher gum levels could be due to this reason as explained below. Primarily, it is considered that starch-gum-water system is a biphasic with the presence of gum in continuous phase (liquid). Soluble molecules are found in the liquid portion at a certain concentration before heating which increases with heating because of the reduction in liquids due to absorption by starch granules. It is considered that concentration of continuous phase shrunk during gelatinization due to amylose leaching from the starch granules and volume of accessible continuous phase for gum was reduced and lead to increase in the concentration of the water soluble molecules. Therefore, the viscosity increases due to the reduction of water molecules caused by starch granules swelling and the increase in the concentration of the water soluble molecules. In other words, we can say that interactions of gum, solubilized starch (leached amylose and shorn chain amylopectin’s) and swollen starch granules resulted in overall higher viscosity of the paste.

The findings of our study are well supported by several previous studies that increase in overall viscosity of the starch hydrocolloid system occurs due to additive effect of the increased viscosity of the hydrocolloid that is more concentrated as the volume, and interaction between the hydrocolloid, solubilized amylose and short chain (low molecular weight) amylopectin’s [31,32,33,34]. As stated above, setback viscosity of the starch gum blends was higher than the plain corn starch. It is reported by von Borries–Medrano, et al. [35] that starches become more susceptible to short term retrogradation in the presence gaur gum. Similar phenomenon could have resulted in the higher setback values of corn starch due to gum *Cordia*, because the gum *Cordia* hydrocolloid is dominated by galactomannan fraction [36]. Short-term retrogradation is affected by several factors including the amylose content, the size of the amylose molecules, and the dispersion of amylose chains in the gel matrix [37,38]. The increase in effective concentration of amylose in the continuous phase could be a possible reason when thickening capacity of the gums (higher water absorption and re)and making the starch-gum system as water deficit system which ultimately restricted the mobility of the amylose, resulting in the ease of interaction between nearby amylose molecules that accelerate short-term retrogradation [39]. The higher setback values are also reported by several researchers when different hydrocolloids were mixed with various starches in different concentrations e.g., [40,41,42]. There was a slight but non-significant decrease in pasting temperature due to the presence of GC in starch blends. Pasting temperature of yam starch was reduced in the presence of guar gum and locust bean gum Huang [43] and xanthan in yam starch [44]. This decrease in pasting temperature could be attributed to the interaction of hydrocolloids with small amount of amylose released by the partial swelling of starch granules [45].

### 3.3. Freeze Thaw Stability of Gels

Frozen food products containing starch are prone to repeated freeze-thaw cycles during storage and transport. The thermal fluctuations during storage and transportation of such foods lead to melting and re-freezing of water that ultimately deteriorates their quality. Starch undergo retrogradation during cooling which can lead to water separation during thawing. Gels obtained from RVA experiment during the present study were subjected to two freeze-thaw cycles at 4 and 8 days after the initial freezing. The separation from gels (irrespective of gum concentration) was higher in 2nd cycle than 1st thaw cycle. It is evident from the data presented in Table 3 that control had the highest (1.06 ± 0.17%) syneresis than all types of gels during first cycle. The syneresis from gels during the 2nd cycle followed a similar trend and was the highest in control (10.02 ± 0.43%). The presence of gum resulted in the reduction in syneresis that could be due to gum interaction with amylose that limited amylose-amylose interaction or binding of extra water from the system. The gradual increase of syneresis with the higher gum concentration could be attributed to the formation of gum-gum regions as compared to gum-amylose regions in the system. Several researchers have reported that presence of hydrocolloid gums improved the freeze-thaw stability of different starches [46,47,48]. The gum *Cordia* can be used as a freeze- thaw stabilizing agent in different types of sauces and frozen products as an alternative to guar, xanthan, agar and locust bean gum with different starches [47,49,50]. The higher syneresis during the 2nd cycle could be attributed to the possibility that once the gels went through thawing and centrifugation during the 1st cycle, their matrix was deteriorated. In a previous study Pongsawatmanit, Chantaro and Nishinari [4], the authors reported a linear increase in the percentage of syneresis (8–20) in tapioca starch-xanthan gum mixtures during 8 freeze-thaw cycles. The results of the present study are well supported by findings of three previous studies Alamri, Mohamed and Hussain [12,13] and Hussain [51] where linseed and okra gum were mixed with rice, corn, and sorghum starches.

### 3.4. Textural Properties of Gels

Gels obtained from RVA experiments were stored overnight at room temperature and TPA studies were conducted. The data pertaining to the TPA properties of gels are presented in Table 4. It is evident from the data that hardness, cohesiveness, adhesiveness, and chewiness of gels were significantly affected by gum levels, while there was no significant change in springiness. The hardness of sample containing 6% gum was the highest (278 ± 4.94 g), while it was the lowest in plain starch (168 ± 14.84 g) among different blends. Hardness of gels could be directly correlated to the retrogradation behavior of starch. The setback values of starch blends determined through RVA experiments (Table 2) represented a similar trend as observed in hardness values. The presence of gums in the system has caused a competition for water between gum and starch components. It is considered that when starches are heated in the presence of gums, the hydrocolloid forms hydrogen bonds with the solubilized starch in swollen starch granules which ultimately reinforces structure made by gum and results in high viscosity starch paste [30]. The crosslinking of gum with amylose and amylopectin present in the continuous phase will also lead to firm gel formation when gel is stored overnight at room temperature. Higher levels of gum (i.e., 9% and 12%) decreased (even though it was higher than control) gel hardness when compared to 6% could be attributed to the starch dilution effect. The dilution effect can be explained as reduction of amylose contents in the blends with increasing the gum level. Higher levels of amylose could result in higher intermolecular crosslinking and vice versa. The gums helps to weaken the amylose networking by impeding between the amylose molecules [52]. Previous studies report that presence of hydrocolloids (linseed, okra, gellan, xyloglucan, glucomannan, and xanthan) increased gel hardness of different starches [13,53,54]. The blends with higher hardness and setback values could be used in ice creams, frozen products, yogurts and other confectionary items [55,56]. Adhesiveness of starch gel decreased as the level of gum in blends increased. The lowest adhesiveness (0.43 ± 0.04 mJ) was observed in the 12% blend, while the highest adhesiveness (1.60 ± 0.14 mJ) was observed in control. Adhesiveness of gels is mainly attributed to the amylose mobility and distribution in starchy foods. In general, it is considered that retrogradation of starch due to amylose association increases hardness and decreases adhesiveness as observed in cooked rice starch [57,58]. The decrease in adhesiveness with the increase in gum concentration during the present study could be attributed to the interactive effect of gum with leached amylose that resulted in increase in hardness and decrease in adhesiveness in corn starch and GC blends. It is reported by Zheng, Sun, Yang, Chen, Liu, Tian and Ye [52] that lower adhesiveness in starchy foods could be a plus point as the gel that doesn’t adheres to lips, teeth and palate is preferred in commercial production. A significant increase in cohesiveness was also noticed in the presence of GC at all levels when compared to control starch (0.40 ± 0.01 mJ). All types of gel texture were solid in appearance; therefore, the chewiness of gels was calculated. The chewiness of gels increased in the presence of gum in blends. The blend with 6% gum had the highest chewiness among all blends. Springiness of the gels is termed as how well the gel physically springs back after it has been deformed during the first compression cycle of texture profile analysis. The gels with less springiness are considered to be less elastic and less rubbery and are easy to masticate [59,60]. The interaction between the dispersed and continuous phase, amylose matrix, and rigidity of starch granules are the determining factors for springiness of gels [28]. It is evident from Table 4 that there was no significant difference among the gels springiness containing different levels of GC. All the gels were elastic enough and no visual deformation was noticed in their overall structure.

### 3.5. Flow Behavior of Starch Gels

The gels obtained from plain corn starch or its blends with GC at various levels were evaluated at 25 °C using a variable shear rate between 0–350 (1/s) to determine the flow behavior. The shear rate (1/s) versus shear stress (σ) data of all samples fitted well to the simple power law model (R^2^ = 0.97–0.99) as shown in Table 5. All samples had flow behavior index (*n*) value of less than 1 which indicates the non-Newtonian (pseudoplastic or shear thinning) behavior. Shear thinning is a phenomenon when the viscosity of the gel is decreased with increasing shear rate. The deviation of value on *n* away from 1 describes that material is more pseudoplastic. The value of *n* was the lowest (0.17) for control starch and it increased to (0.36–0.56) at different levels of gums in starch. The data indicates that presence of gum had resulted in reduction in pseudoplasticity when compared to control. The lower degree of psuedoplasticity in the presence of GC could be attributed to the formation of intra-molecular hydrogen bonding in gum molecules, which resulted in the shortage of hydroxyl groups to form inter-molecular hydrogen bonding with amylose. Several researchers have reported the decrease in pseudoplastic behavior of different starches in the presence of locust bean gum and Tara gum [17,61] while others have reported an increase in the psuedoplasticity of different starches due to the presence of linseed, okra, and xanthan gums [13,51,62]. It is evident from the results that though all the gels were pseudoplastic in nature and were elastic enough to be used in commercial production where easy swallowing, less stickiness with teeth’s and lips is required, e.g., ice creams, whipped creams, ketchups and other frozen desserts.

The consistency index (*K*) is directly related with the viscosity of starch gels, which increased with the increase in concentration of GC. The value of *K* was higher in all blends (0.69–0.76 Pa s^n^) as compared to control (0.65 Pa s^n^) which represents synergism between gum and starch. The *K* value is directly related to thickness of the samples and the results of viscosity observed in RVA experiments (Table 2) are correlated with the results presented in Table 5. The starch systems are considered to be composite systems in which amylopectin is dispersed during the continuous phase (amylose). Therefore, the presence of gum in the continuous phase results in reduction of volume and a lower availability of water which ultimately increases the thickness. It further reflects the excellent hydration capability of GC in corn starch system. The results of present study are consistent with many other studies, e.g., [17,61,63], which reported increased values of consistency indices of wheat, okra, rice, and corn starches in the presence of locust gum, flaxseed, and Tara gum.

### 3.6. Dynamic Shear Properties of Starch Gels

**Figure 1:**
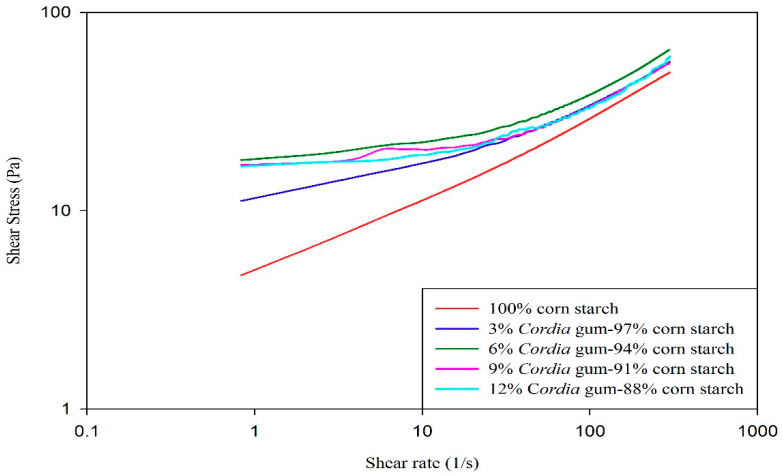
Effect of gum *Cordia* on the flow curves (shear stress) of corn starch.

**Figure 2:**
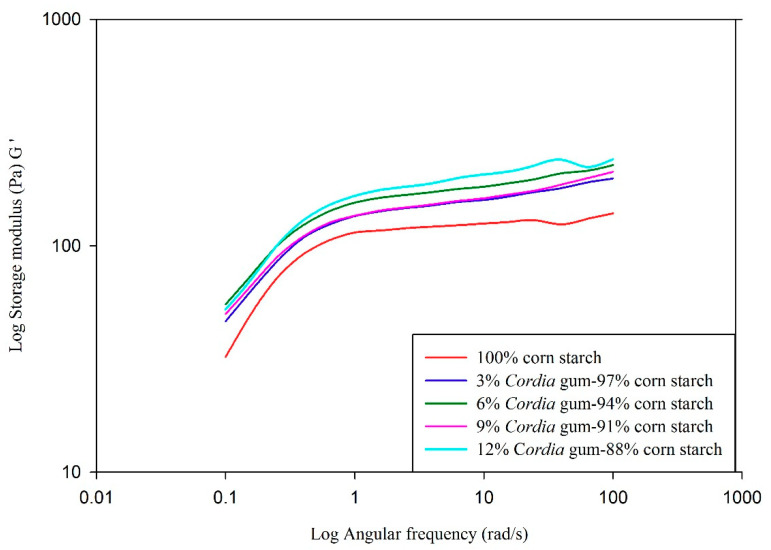
Effect of gum *Cordia* on the storage (elastic) modulus (G′) of corn starch gels.

**Figure 3:**
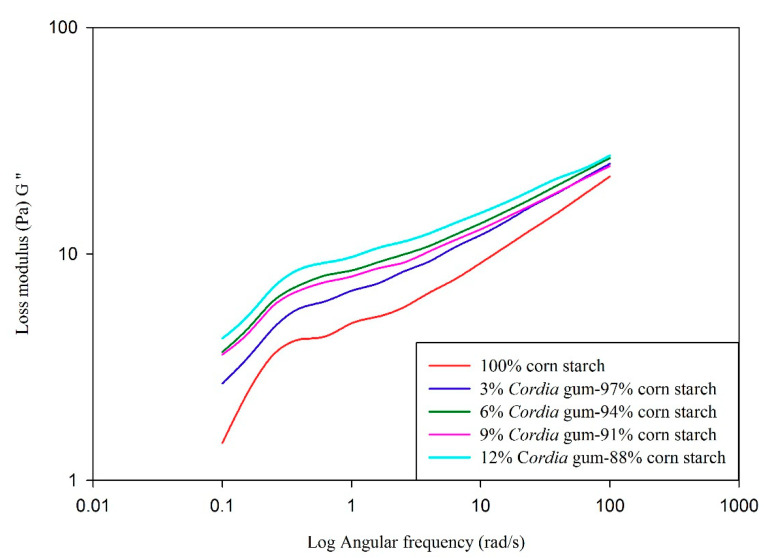
Effect of gum *Cordia* on the loss modulus (G″) of corn starch gels.

**Figure 4:**
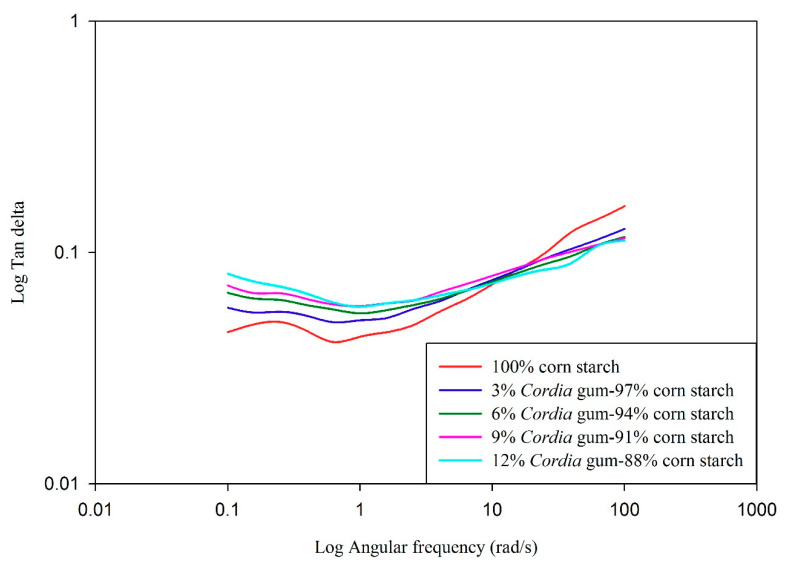
Effect of gum *Cordia* on the tan delta (δ) of corn starch gels.

Data relating to storage/elastic modulus (G′), loss/viscous modulus (G″) and tan delta (δ, G″/G′) as a function of angular frequency (rad/s) is presented in Figure 1, Figure 2, Figure 3 and Figure 4, respectively. The elastic/storage modulus (G′) is a measure of the energy that is stored in the material or recoverable per cycle of deformation whereas, loss/viscous modulus (G″) is the measure of energy which is lost as viscous indulgence per cycle of distortion of material. The storage or elastic modulus (G′) gives information about the elastic structure and solid like (energy stored) properties of the material while loss or viscous modulus (G″) represents the viscous character and liquid like (energy dissipated) properties. It is evident from the data presented in graphs that, the value of G′ was much higher than G″, thus showing the dominance of elastic properties over viscous properties. If the value of storage modules is higher than loss modulus, the material can be considered as mainly elastic [64]. The prediction of viscoelastic behaviors can also be evaluated by the value of tan δ (G″/G′). The value of tan < 1 represents elastic behavior, while ˃1 indicates viscous behavior. The value of control was higher (closer to 1) in comparison to blends with gums (closer to 0) indicating a higher elasticity in gum blends. Overall, both the viscous and elastic properties of all blends were increased as a function of angular frequency. The values of G′ and G″ were frequency dependent with no crossover between the two moduli. The magnitudes of G′ and G″ were also dependent on gum concentration and increased as the gum concentration increased. The values of both moduli were the highest in the blend containing 12% GC as compared to plain starch (control). There was a strong relationship between gum and starch where higher angular frequency signifies the greater elasticity (G′ > G″) and vice versa. Similar trend was also reported in rice starch-xanthan gum [61], tapioca starch-xanthan gum [14], and waxy corn-galactoxyloglucan [65] mixtures. The data tabulated in Table 6 represents G′, G″, and δ values of corn starch-gum blends at 6.3 rad/s. The values of G′, G″, and δ increased with the increase in gum (0 to 12%) concentration. The increase in value of δ with increasing the GC in blends can also be correlated with the peak viscosity measured through RVA and *K* value presented in Table 5 which indicates increase in viscous behavior of gel as function of gum level. Higher values of dynamic moduli can be attributed to the more viscoelastic behavior of gums at higher concentrations. Increase in the moduli could be attributed to the presence of gum in continuous phase of starch gum system [66]. This clearly reveals the synergistic effect of gum with starch which is apparent when we compare the G′ of control (123) with that of blends (156–199). The outcomes of the current study are consistent with sweet potato starch-xanthan, flaxseed, cress seed, okra [67] corn-xanthan [66] and rice starch-galactomannan [61] mixtures.

## 4. Conclusions

Gum *Cordia* (GC) is a natural polysaccharide and can be easily extracted from the mature *Cordia* fruit. The gum was used to modify the corn starch properties so that it can be used as an alternative to the other commonly used hydrocolloids, e.g., xanthan, gum Arabic, guar gum, etc. Significant increases in the starch gelatinization temperatures, enthalpies, peak viscosities, final viscosities, and setback viscosities were observed in the presence of gum *Cordia*. The flow behavior index (*n*) value of all samples was less than one, indicating the non-Newtonian (pseudoplastic or shear thinning) behavior. The consistency index (*K*) of the starch-gum blends increased with higher levels of gum *Cordia*. The magnitudes of G′ and G″ were also dependent on gum concentration and increased as a function of gum concentration. The presence of gum m resulted in the reduction in syneresis that could be due to gum interaction with amylose. Hardness, cohesiveness, adhesiveness, and chewiness of gels were significantly affected in the presence of *Cordia* gum. These results can be used in formulation of corn starch-GC based products with improved textural, rheological and stability properties.

## Figures and Tables

**Table 1:** DSC (differential scanning calorimeter) properties of corn starch as a function of different levels of gum *Cordia*.

	0%	3%	6%	9%	12%
**ΔH (J/g)**	12.08 ± 0.19d	12.97 ± 0.07c	13.26 ± 0.11b	13.63 ± 0.10a	11.86 ± 0.06e
**OT (°C)**	66.34 ± 0.15e	68.66 ± 0.15d	70.13 ± 0.20c	70.62 ± 0.08b	71.34 ± 0.12a
**PT (°C)**	70.96 ± 0.52e	74.03 ± 0.09d	75.22 ± 0.11c	76.61 ± 0.20b	77.66 ± 0.15a

Abbreviations: PT = peak temperature, OT = onset temperature. Means that do not share a letter within a row are statistically significant (*p* < 0.05).

**Table 2:** RVA (rapid visco analyzer) properties of corn starch as a function of different levels of gum *Cordia*.

Sample	PV (cP)	Trough (cP)	BD (cP)	FV (cP)	SB (cP)	Peak Time (Min)	PT (°C)
0%	2002 ± 24.04d	921 ± 42.43d	1081 ± 18.38d	1915 ± 39.60d	994 ± 2.83e	5.07 ± 0.00a	75.93 ± 0.11a
3%	2911 ± 34.65c	1118 ± 33.23c	1793 ± 1.41c	2558 ± 12.20c	1441 ± 33.23d	4.74 ± 0.09b	74.50 ± 0.07b
6%	3134 ± 28.28b	1110 ± 34.65c	2025 ± 62.93c	2986 ± 20.51a	1876 ± 14.14a	4.57 ± 0.05c	74.08 ± 0.18b
9%	3234 ± 26.16a	1182 ± 18.39bc	2052 ± 44.55bc	2894 ± 2.12c	1712 ± 20.51b	4.57 ± 0.05c	74.20 ± 0.07b
12%	3269 ± 33.23a	1338 ±7.78a	1931 ± 25.46a	3032 ± 15.56a	1695 ± 7.78c	4.60 ± 0.00c	74.28 ± 0.04b

Abbreviations: PV = peak viscosity; BD = breakdown; FV = final viscosity; SB = setback; PT = peak temperature; cP = centipoise. Means that do not share a letter within a column are statistically significant (*p* < 0.05).

**Table 3:** Percentage syneresis from the cornstarch gels as a function of gum *Cordia*.

Sample	1st 4 Days	2nd 4 Days	Total
0%	1.06 ± 0.17a	10.02 ± 0.43a	11.08 ± 0.60a
3%	0d	0.84 ± 0.11e	0.84 ± 0.11e
6%	0d	1.52 ± 0.09d	1.52 ± 0.09d
9%	0.12 ± 0.02c	4.73 ± 0.07c	4.85 ± 0.09c
12%	0.30 ± 0.03b	7.67 ± 0.50b	7.97 ± 0.53b

Means that do not share a letter within a column are statistically significant (*p* < 0.05).

**Table 4:** TPA (texture profile analysis) properties of starch gels as a function of gum *Cordia*.

Sample	Hardness (g)	Cohesiveness	Springiness (mm)	Adhesiveness (mJ)	Chewiness (g.mm)
0%	168 ± 14.84a	0.40 ± 0.01e	10.15 ± 0.07a	1.60 ± 0.14a	683 ± 40.86e
3%	231 ± 8.48d	0.44 ± 0.02d	10.3 ± 0.14a	1.05 ± 0.07b	1034 ± 1.74d
6%	278 ± 4.94b	0.52 ± 0.00a	10.15 ± 0.07a	0.75 ± 0.07c	1479 ± 3.84a
9%	245 ± 4.96c	0.49 ± 0.01b	10.1 ± 0.03a	0.60 ± 0.00d	1210 ± 10.43b
12%	233 ± 2.12d	0.48 ± 0.00c	10.25 ± 0.07a	0.43 ± 0.04e	1144 ± 18.33c

Means that do not share a letter within a column are statistically significant (*p* < 0.05).

**Table 5:** Effect of *Cordia* gum on the consistency coefficient (*K*) and flow behavior index (*n*) of corn starch.

	0%	3%	6%	9%	12%
***n***	0.17 ± 0.01e	0.36 ± 0.02d	0.41 ± 0.05c	0.56 ± 0.03a	0.50 ± 0.01b
***K*** (Pa s^n^)	0.65 ± 0.01c	0.69 ± 0.05b	0.72 ± 0.02b	0.75 ± 0.06a	0.76 ± 0.02a
**R^2^**	0.97 ± 0.01a	0.98 ± 0.01a	0.99 ± 0.03a	0.98 ± 0.03a	0.98 ± 0.02a

Means that do not share a letter within a row are statistically significant (*p* < 0.05).

**Table 6:** Storage modulus G′, Loss modulus G″ and δ at 6.3 rad/s of corn starch-gum blends.

	0%	3%	6%	9%	12%
**G′ (Pa)**	123 ± 2.3	156 ± 4.6	178 ± 3.4	157 ± 5.2	199 ± 2.2
**G″ (Pa)**	7.72 ± 0.4	10.70 ± 0.2	12.15 ± 0.4	11.49 ± 0.7	13.68 ± 0.5
**Tan delta (δ)**	0.062	0.068	0.068	0.073	0.069
